# Zika Inquiries Made to the CDC-INFO System, December 2015–September 2017

**DOI:** 10.3201/eid2605.181694

**Published:** 2020-05

**Authors:** Tara Kirk Sell, Crystal Watson, Diane Meyer, Michael R. Snyder, Sanjana J. Ravi, Emma E. McGinty, Laura E. Pechta, Dale A. Rose, Michelle N. Podgornik, Keri M. Lubell

**Affiliations:** Johns Hopkins Bloomberg School of Public Health, Baltimore, Maryland, USA (T.K. Sell, C. Watson, D. Meyer, M.R. Snyder, S.J. Ravi, E.E. McGinty);; Centers for Disease Control and Prevention, Atlanta, Georgia, USA (L.E. Pechta, D.A. Rose, M.N. Podgornik, K.M. Lubell)

**Keywords:** CDC-INFO, communication, disease outbreak, geolocation, information seeking, sexually transmitted infections, vector-borne infections, viruses, Zika, Zika testing, Zika transmission, Zika virus

## Abstract

We examined Zika-related inquiries to CDC-INFO, the national contact center for the Centers for Disease Control and Prevention, to identify potential communication gaps. The most frequently asked questions related to travel or geographic location of Zika (42% of all inquiries), information about laboratory testing (13%), or acquiring a Zika test (11%).

A rapid increase in Zika virus disease transmission rates throughout Latin America in 2015, followed by transmission in some US states and outbreaks in several US territories, sparked widespread attention (*1*–*3*). We systematically examined Zika-related inquiries to the CDC-INFO system, the national contact center for the Centers for Disease Control and Prevention to determine public concerns and questions about Zika and potential communication gaps. We analyzed inquirer type, inquiry topic, and number of inquiries. In this article, “question” refers to the content of each call/email and “inquiry” to individual calls/emails, regardless of content. Inquiries may include >1 question. 

The CDC-INFO Zika dataset contained 32,668 English-language inquiries (calls/emails) about Zika made from December 1, 2015 (when inquiries about Zika began to be tracked), through September 29, 2017 (when CDC’s emergency activation for Zika response ended). We analyzed the number of inquiries over time using all database records and information on inquirers and topics using a 10% simple random sample (n = 3,268). After an initial pilot process, 2 study authors coded notes made by operators for information on the types of inquirers and types of questions (*4*) (Appendix).

We grouped inquirers into 3 different categories; however, most did not specify a category affiliation. The first category, the pregnancy group, made up 19% of all inquirers and was composed of pregnant women (10%), women planning to become pregnant and their partners (6%), and partners of pregnant women (3%). The second category, clinicians, made up 14% of all inquirers. (CDC also initiated a separate hotline for clinicians during the Zika response; information from those calls was not included in this data analysis.) The third category, all other inquirers (67%), included family members and parents (8%); public health practitioners, students and educators, politicians and political staff, media, and salespeople (3%); and inquirers who did not specify identity (56%). 

The most frequent questions (present in 42% of inquiries) were about travel or geographic location (geolocation) of Zika outbreaks (Table). Approximately 13% of all inquiries included questions seeking factual background information about laboratory testing, including how to obtain results, how to administer tests, how to interpret results, and criteria for testing. Questions about getting tested for Zika were present in 11% of inquiries. Questions related to transmission factors (e.g., incubation, persistence, immunity, semen, mosquitoes) were present in 9% of inquiries. Only 4% of inquiries included questions about health effects and related issues (e.g., potential harm to fetus, self, children) (Table). 

**Table:** Percentage of inquiries with specific question topics to CDC-INFO, December 1, 2015–September 29, 2017*

Question topic	% Inquiries with question topic, n = 3268
Information gathering	
Transmission: includes persistence, presence in semen, mosquitoes, and immunity	9
Incubation period	<1
Signs and symptoms	2
Outbreak response processes	3
Seeking information about diagnostic tests	13
Seeking information about treatments, countermeasures, vaccines	1
Clinician seeking clinical recommendation/assistance for a patient with Zika	1
Information about risks	
Health effects/issues: includes harm to self, fetus, pregnant woman, or child	4
Health effects: specifically long-term reproductive effects	4
Exposure: mosquito-related or sexual exposure	2
Infection: asking if inquirer could have Zika	1
Safety of protective actions: includes spraying or repellant	<1
Actions	
Protective actions	
What activities should be done to protect from getting Zika	3
Waiting to get pregnant	4
Safe sex practices	1
Insect repellent/preventing bug bites/mosquito control	3
What action to take following possible exposure	1
Acquiring a Zika test	11
Travel and geolocation	42
Actions for infected persons	<1
Other	
Seeking access to materials/tools	4

*When inquirers asked >1 type of question, each type was counted separately. As a result, total percentage adds up to >100%. CDC-INFO, the national contact center for the Centers for Disease Control and Prevention.

Some types of questions were asked more frequently than others by certain groups. For example, the pregnancy group most frequently asked questions about travel or geolocation of the disease (65% of all inquiries from this group), whereas clinicians most frequently sought information about tests (46%) (Appendix Table 1). 

Analysis of the number of inquiries over time showed 2 distinct peaks. The first occurred early in the response, with ≈4,000 biweekly inquiries at the peak in late January/early February 2016, when news media coverage of the outbreak increased. The second occurred in late July/early August 2016, after local US transmission was confirmed, with nearly 2,000 biweekly inquiries (Appendix Figure 1). The number of inquiries by date for the 4 most frequently asked questions (about travel or geolocation of Zika, seeking information about tests, seeking to be tested for Zika, and transmission) generally reflected the same overall pattern as all inquiries. One exception was questions about travel/geolocation of the disease, which showed a third, smaller rise in volume in late 2016/early 2017 (Appendix Figure 2). The frequency of questions about transmission, signs/symptoms, health effects, long term health effects, and insect management was significantly greater early in the outbreak, before local transmission was confirmed in the United States (p<0.05 by χ^2^ test). Questions about waiting to get pregnant and geolocation were made significantly more often after local transmission occurred (p<0.05 by χ^2^ test).

Outreach to CDC-INFO might indicate that Zika messages reached intended target populations. Of those who volunteered demographic information, 44% were in the pregnancy group and so might have been made aware of heightened risks and sought more specific information about ways to reduce them. Results show that information needs were most intense at the time the threat emerged, although events during the outbreak, such as news of local transmission cases in the United States or new transmission routes, might also have increased public interest. 

Inquiries made to CDC-INFO about Zika might represent potential information gaps from other sources. Inquirers most frequently asked about travel/geolocation of disease and testing, which was already included in CDC messaging and disseminated through various channels. Our findings could indicate that the information previously provided was perceived to be insufficient or difficult to locate or understand or that it was hard to keep up with changes in messaging during the response. 

CDC-INFO records are a source of data that has been underanalyzed but that provides critical information about inquiries from the public about active efforts to obtain information from CDC. Our findings may help with future messaging efforts around infectious disease outbreaks by identifying topics of information that should be emphasized to improve public understanding. 

Appendix. Methodology for sampling, content analysis and measures, and data analysis.
