# Supplementary material for: Zika Inquiries Made to the CDC-INFO System, December 2015–September 2017
Source: Emerg Infect Dis. 2020 May;26(5):1022–4. doi: 10.3201/eid2605.181694 (PMC7181940; doi:10.3201/eid2605.181694)
Supplement: Supplementary file 1 — Appendix. Methodology for sampling, content analysis and measures, and data analysis. [file 18-1694-Techapp-s1.pdf]

Article DOI: https://doi.org/10.3201/eid2605.181694

Zika Inquiries Made to the CDC-INFO System, December 2015–September 2017

Appendix

Methods

Terminology

The term “question” is used to specify content of each call or email to CDC-INFO while “inquiry” is used as an umbrella term to describe calls or emails to CDC-INFO, regardless of specific content, and may include >1 question.

Sample

The full CDC-INFO Zika dataset contained 32,668 English language inquiries (calls and emails combined) made from December 1, 2015 (when inquiries about Zika began to be tracked) to September 29, 2017 (when the CDC’s emergency activation for Zika response ended). These inquiries were identified using a combination of a keyword search on the term “Zika” and a search on a list of Zika prepared responses identification numbers that operators log when responding to an inquiry. Each inquiry was recorded separately, even if it was made by an individual who had previously contacted CDC-INFO. To evaluate public inquiries about Zika, we analyzed a 10% simple random sample (N = 3,268).

Content Analysis Approach and Measures

The research team reviewed the information gathering process specific to the general public described by Slaughter et al. (*1*) and developed a coding instrument that focused on general health-related information gathering (i.e., questions addressing general knowledge gaps about Zika), questions about Zika-related risks, and questions about potential actions to be taken. Emotional and social themes (e.g., fears/concerns, pressure from family) were considered but were determined to be too difficult to reliably interpret using the notes of CDC-INFO agents.

Five members of the research team piloted the coding instrument on a random sample of 350 inquiries that were not included in the final analytic sample. Piloting was performed to test

the coding instrument, note potential issues, ensure group consensus on the meaning of codes, and revise the instrument. The team iteratively reviewed the instrument after each round of 50 pilot inquiries. The final coding instrument included date of inquiry, type of interaction, type of inquirer, and items related to potential questions about the Zika virus (Appendix Table 2). Type of inquirer was coded from the question text and the self-identification field. If an inquiry included >1 question, each question type was included.

Two members of the team independently coded the first 600 inquiries of the analytic sample to assess interrater reliability for each question on the instrument, coded as a dichotomous (yes/no) variable. Kappa values and percent agreement were then calculated on the final set of coded items (N=22). All coding items (i.e. Zika question topics) had high percent agreement, with the majority showing greater than 99% agreement and the lowest item showing 96% agreement. The majority of coding items had kappa values above 0.80, and all but 1 coding item/grouping had kappa values above 0.69. (*2*) This item, related to misinformation, was found to be too subjective and was excluded from the final analysis (Appendix Table 3). The remainder of the sample (N=2,668) was split evenly between coders, who periodically reviewed difficult-to-interpret questions with 2 research team members to ensure continued agreement.

Data Analysis

Descriptive statistics related to volume and inquiry type (phone call or email) were calculated using the full CDC-INFO Zika dataset. The 10% random sample described above was used for the remainder of the analysis. To evaluate the content of inquiries to CDC-INFO, we calculated the proportion of inquiries in the sample that included each Zika-related question. Chi-squared tests were used to test differences in question frequency before and after local disease transmission was confirmed in the continental United States.

References

1. Slaughter L, Keselman A, Kushniruk A, Patel VL. A framework for capturing the interactions between laypersons' understanding of disease, information gathering behaviors, and actions taken during an epidemic. J Biomed Inform. 2005;38:298–313. PubMed https://doi.org/10.1016/j.jbi.2004.12.006

2. Landis JR, Koch GG. The measurement of observer agreement for categorical data. Biometrics. 1977;33:159–174.

Appendix Table 1. Percentage of inquiries with question topic by three types of inquirers to CDC-INFO, December 1, 2015–September 29, 2017

Question topic	% of pregnancy group* (n=624)	% of clinicians (n=443)	% of all other inquirers (n=2201)
Information gathering			
Transmission (Includes persistence, presence in semen, mosquitoes, and immunity)	6	5	11
Incubation period	<1	<1	<1
Signs and symptoms	<1	<1	3
Outbreak response processes	2	3	3
Seeking information about diagnostic tests	6	46	8
Seeking information about treatments, countermeasures, vaccines	<1	1	2
Clinician seeking clinical recommendation/assistance for a patient with Zika	0	5	0
Information about risks			
Health effects/issues (includes harm to self, fetus, pregnant woman, or child)	3	2	4
Health effects, specifically long-term reproductive effects	<1	1	5
Exposure (mosquito-related or sexual exposure)	3	1	2
Infection (Asking if inquirer could have Zika)	1	0	1
Safety of protective actions (Includes spraying or repellant)	<1	<1	<1
Actions			
Protective actions: what activities should be done to protect from getting Zika	3	0	3
Protective actions: waiting to get pregnant	6	4	3
Protective actions: safe sex practices	2	1	<1
Protective actions: insect repellent/prevention bug bites/mosquito control	2	<1	3
Protective actions: what action to take following possible exposure	1	<1	1
Acquiring a Zika test	14	12	9
Travel and geolocation	65	12	41
Actions for infected persons	0	<1	<1
Other			
Seeking access to materials/tools	<1	9	5

* Inquirers who were pregnant, the partners of pregnant women, or those who were planning on starting a family

Appendix Table 2. Coding topics, percent agreement for coders, and interrater reliability as measured by Kappa Scores

Question topic	% agreement	Kappa
Transmission (Includes persistence, presence in semen, mosquitoes, and immunity)	98.8	0.92
Incubation period	100.0	1.00
Signs and symptoms	100.0	1.00
Outbreak response processes	98.5	0.70
Seeking information about diagnostic tests	97.0	0.85
Seeking information about treatments, countermeasures, vaccines	99.8	0.94
Clinician seeking clinical recommendation/assistance for a patient with Zika	99.7	0.75
Health effects/issues (includes harm to self, fetus, pregnant woman, or child)	97.2	0.74
Health effects, specifically long-term reproductive effects	99.7	0.83
Exposure (mosquito-related or sexual exposure)	99.8	0.80
Infection (asking if inquirer could shave Zika)	99.7	0.80
Safety of protective actions (Includes spraying or repellant)	99.9	0.86
Protective actions: what activities should be done to protect from getting Zika	98.8	0.78
Protective actions: waiting to get pregnant	99.5	0.91
Protective actions: safe sex practices	99.5	0.72
Protective actions: insect repellent/prevention bug bites/mosquito control	99.9	0.89
Protective actions: what action to take following possible exposure	100.0	1.00
Acquiring a Zika test	96.3	0.79
Travel and geolocation	96.0	0.92
Actions for infected persons	100.0	1.00
Seeking access to materials/tools	98.0	0.84
Questions/comment about rumors or misinformation	99.5	0.40

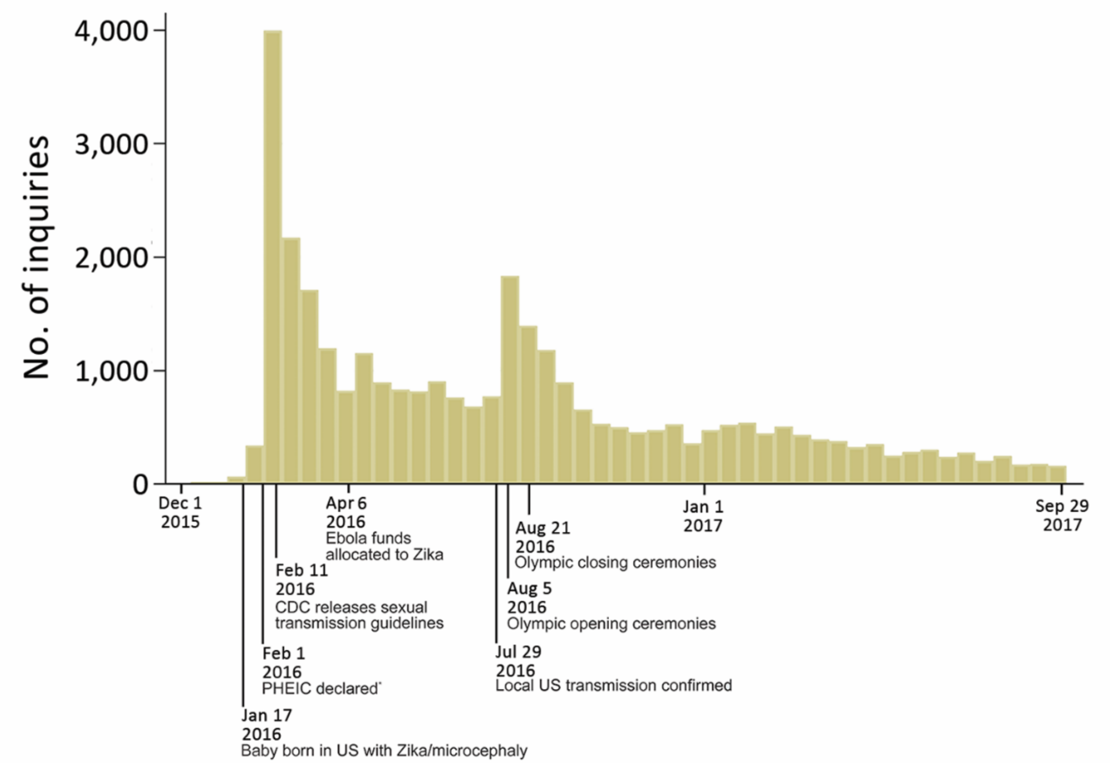

Appendix Figure 1. Number of CDC-INFO Zika inquiries over time December 1, 2015–September 29, 2017. Total inquiries: 32,668. Each bar represents a 14-day period. *PHEIC: Public Health Emergency of International Concern.

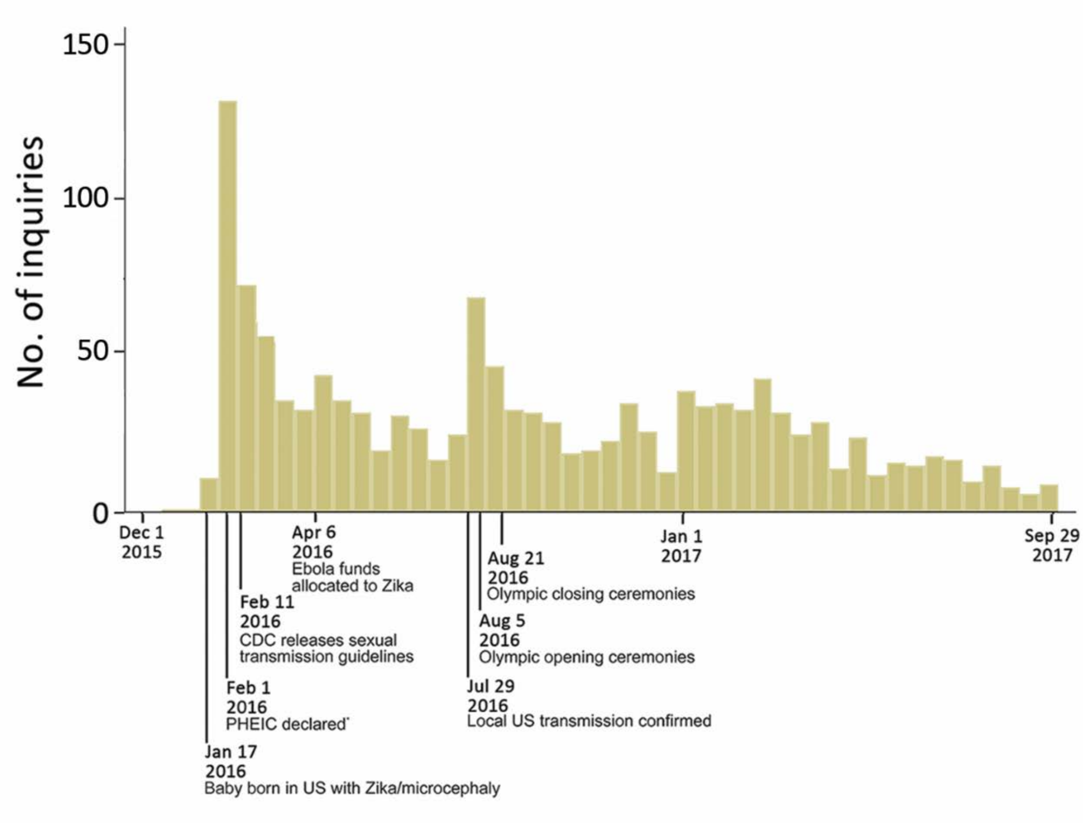

Appendix Figure 2. Inquiries to CDC-INFO about travel and geolocation of Zika over time for all inquirers December 1, 2015 – September 29, 2017. Total inquiries: 32,668. Each bar represents a 14-day period. *PHEIC: Public Health Emergency of International Concern.
